# Outcomes in adulthood after neurosurgical treatment of brain tumors in the first 3 years of life: long-term follow-up of a single consecutive institutional series of 97 patients

**DOI:** 10.1007/s00381-021-05130-x

**Published:** 2021-06-11

**Authors:** Tryggve Lundar, Bernt Johan Due-Tønnessen

**Affiliations:** 1 Department of Neurosurgery, Oslo University Hospital, Postboks 4950 Nydalen, 0424 Oslo, Norway; 2 Department of Neurosurgery, Faculty of Medicine, University of Oslo, Oslo, Norway

Dear Editor:

We thank Steinbok and Pillai for their response to our paper “Outcomes in adulthood after neurosurgical treatment of brain tumors in the first 3 years of life: long-term follow-up of a single consecutive institutional series of 97 patients” [1]. They express compliments but also regret that their paper from 2012 was not listed in our references [2].

I was not aware of their paper, and agree that their publication as well as ours shows major differences in outcome between children with high-grade and low-grade tumors. Since their paper presents results in children in their first year of life, this reference could and perhaps should have been included in our previous publication in 2019, analyzing this specific age group [3].

As described in the introduction of our recent paper, our 97 cases include both infants under the age of 6 months and in their first year of life, in addition to those in their second and third year of life, all with follow-up of at least 20 years. Some have argued that this was a kind of double publication. We waited until all children had 20 years follow-up, and therefore have full datasets on 20 years *observed survival*.

As most publications also include patients with short follow-up, Kaplan-Meier survival plots may be illusive as the right part of the plots is based on few individuals. The use of *observed survival rates*, as we suggest, will therefore be more accurate. In our recent paper, all 20-year survivors are 20 years and above, allowing implementation of valuable information about long-term outcome also in terms of schooling and education as well as participation in part-time and full-time work.

The extended follow-up gives insight into the profound changes in prognosis over time for small children with brain tumors. This experience argues itself for repeated surgical resections instead of adjuvant radiotherapy when feasible.

With kind regards

Tryggve Lundar
